# [Retracted] MSK1 downregulation is associated with neuronal and astrocytic apoptosis following subarachnoid hemorrhage in rats

**DOI:** 10.3892/ol.2026.15520

**Published:** 2026-03-09

**Authors:** Bo Ning, Geng Guo, Hong Liu, Lei Ning, Bao-Liang Sun, Zhen Li, Shuo Wang, Zheng-Wen Lv, Cun-Dong Fan

Oncol Lett 14: 2940–2946, 2017; DOI: 10.3892/ol.2017.6496

Following the publication of the above paper, it was drawn to the Editor's attention by a concerned reader that both the ‘Control’ and ‘SAH (3 days)’ data panels (100× magnification) showing the results of immunohistochemical staining experiments in Fig. 3 on p. 2943 exhibited apparent anomalies, namely the repeated patternings of small areas of cellular data within the same figure panels.

Owing to the potentially anomalous presentation of data in Fig. 3, the Editor of *Oncology Letters* has decided that this paper should be retracted from the Journal. The authors were asked for an explanation to account for these concerns, but the Editorial Office did not receive a satisfactory reply. The Editor apologizes to the readership for any inconvenience caused.

